# Association of age with the non-achievement of clinical and functional remission in rheumatoid arthritis

**DOI:** 10.1038/s41598-020-72274-2

**Published:** 2020-09-17

**Authors:** Tadashi Aoki, Hideki Ito, Takehisa Ogura, Ayako Hirata, Yuji Nishiwaki, Hideto Kameda

**Affiliations:** 1 Division of Rheumatology, Department of Internal Medicine, Graduate School of Medicine, Toho University (Ohashi Medical Center), 2-22-36 Ohashi, Meguro-ku, Tokyo, 153-8515 Japan; 2 Department of Environmental and Occupational Health, Faculty of Medicine, Toho University, Tokyo, Japan

**Keywords:** Rheumatology, Musculoskeletal abnormalities, Rheumatoid arthritis

## Abstract

Patient-reported outcome (PRO) is included in the remission criteria of rheumatoid arthritis (RA). We aimed to determine the effect of age on PRO and the subsequent achievement of clinical and functional RA remission criteria. Three hundred and one patients with non-rheumatic diseases were evaluated using the 0–10 cm visual analog scale (VAS) assessment for musculoskeletal symptoms and a functional health assessment questionnaire-disability index (HAQ-DI). These assessments were compared with those obtained from 149 patients with RA with negative tender/swollen joint counts and normal serum C-reactive levels (objective clinical remission). Of the 301 patients, 32.2%, 26.6%, and 41.2% were classified as non-elderly (< 65 years), early elderly (65–74 years), and late-elderly (≥ 75 years) patients, respectively. VAS > 1 cm and HAQ-DI ≥ 0.5 were observed in 7.3% and 14.5%, respectively, in late-elderly patients, whereas ≤ 1.0% of non-elderly and early elderly patients for the both. Among 149 RA patients in objective remission, however, > 20% and > 10% of early elderly patients (and even non-elderly patients) had VAS > 1 cm and HAQ-DI ≥ 0.5, respectively, and 34.0% and 35.8% of late-elderly patients with RA had VAS > 1 cm and HAQ-DI ≥ 0.5, respectively. Multivariate logistic analysis revealed that age and RA were associated with the non-achievement of VAS ≤ 1 cm and HAQ-DI < 0.5. Therefore, the effect of age, which was independent of the presence of RA even without any objective disease activity, on PRO and the non-achievement of clinical and functional remission criteria was demonstrated.

## Introduction

Patient-reported outcome (PRO) is an important clinical measure of rheumatoid arthritis (RA) disease activity, which is characterized by persistent and eventually destructive polyarthritis^1,2^. However, PRO measures such as the global assessment of disease activity in which the patient uses a 10-cm (100-mm) visual analog scale (VAS) may be influenced by factors other than RA disease activity such as joint damage, osteoarthritis, and psychological distress^3^. Patient VAS has often been reported to be a limiting factor for reaching remission^4^. Even for subjective symptoms of individual joints, the concordance of joint symptoms determined by ultrasound-defined synovitis was more limited than joint swelling determined by rheumatologists^5^, although the assessment of joint symptoms by patients themselves was sensitive, and, thus, may be useful as a screening tool for the presence of arthritis.

The above mentioned strengths and weaknesses of PRO have effects on designating the clinical remission of RA, which has been defined by the American College of Rheumatology (ACR)/The European League Against Rheumatism (EULAR). Remission criteria, whether using the Boolean-based or composite measure simplified disease activity index (SDAI) score, are composed of the following components: tender joint count (TJC), swollen joint count (SJC), patient VAS, serum C-reactive protein (CRP) levels and, in addition, a physician’s global assessment of disease activity using SDAI scores alone^6^. We have recently reported that ~ 50% of patients with RA achieved clinical remission as defined by the SDAI scores, and that advanced age negatively affected remission achievement irrespective of objective findings such as inflammatory joint counts^7^. However, a study that only included patients with RA may not be sufficient for determining the effect of age on PRO in RA. A previous report^8^ suggested that the majority of individuals above 50 years of age did not meet the previous version of ACR remission criteria^9^. The patients’ age was shown to be an important factor affecting the health assessment questionnaire-disability index (HAQ-DI) and patient VAS scores^10,11^. Therefore, we conducted a cross-sectional study comparing 301 patients without defined rheumatic disease with RA patients with negative joint counts and normal serum CRP levels experiencing “objective” clinical remission to further delineate the relevance of age on PRO measures such as patient VAS and HAQ-DI scores.

## Results

### Patient characteristics

Of the 301 patients without rheumatic diseases involved in the study, 54.8% were female (Table 1). The median age of the group that did not have rheumatic disease was 70 years, and the proportion of non-elderly (< 65 years), early elderly (65–74 years), and late-elderly (≥ 75 years) were 32.2%, 26.6%, and 41.2%, respectively. Major diagnoses of non-rheumatic diseases of the group included hypertension (40.2%), cervical atherosclerosis (25.2%), chronic gastritis (54.5%), and hyperlipidemia (48.2%).

**Table 1:** Characteristics of patients without rheumatic diseases (n = 301).

Female sex, n (%)	165	(54.8)
Age, median (IQR)	70	(62–81)
< 65 years, n (%)	97	(32.2)
65–74 years, n (%)	80	(26.6)
≥ 75 years, n (%)	124	(41.2)
Major comorbidities (n ≥ 10)		
Cardiovascular disorders		
Hypertension, n (%)	121	(40.2)
Atrial fibrillation, n (%)	15	(5.0)
Angina, n (%)	14	(4.7)
Cerebrovascular disorders		
Cervical atherosclerosis, n (%)	76	(25.2)
Chronic cerebral ischemia, n (%)	36	(12.0)
Gastrointestinal disorders		
Chronic gastritis, n (%)	164	(54.5)
Barrett’s esophagus, n (%)	45	(15.0)
Reflux esophagitis, n (%)	40	(13.3)
Gastric ulcer, n (%)	23	(7.6)
Cholelithiasis, n (%)	26	(8.6)
Constipation, n (%)	22	(7.3)
Metabolic disorders		
Hyperlipidemia, n (%)	145	(48.2)
Hyperuricemia, n (%)	34	(11.3)
Diabetes mellitus, n (%)	29	(9.6)
Neuropsychiatric disorders		
Dementia, n (%)	15	(5.0)
Respiratory disorders		
Bronchial asthma, n (%)	10	(3.3)
Urogenital disorders		
Prostate hypertrophy, n (%)	11	(3.7)

Values are reported as total number (percentage) or median values (interquartile range; IQR).

Of the 304 patients with RA considered, 149 (49.0%) had no tender or swollen joints and normal serum CRP values (≤ 0.3 mg/dl), namely “objective clinical remission”. Of these, 73.8% were women (Supplementary Table 1). The median age of the patients with RA having negative joint counts and normal CRP values was 72 years, and the rates of non-elderly (< 65 years), early elderly (65–74 years), and late-elderly (≥ 75 years) were 32.2%, 32.2%, and 35.6%, respectively. Therefore, patients with RA had a comparable age distribution to that of non-rheumatic patients, although predominance of women patients was observed in patients with RA, as expected. Compared with the remaining patients with RA, those with negative joint counts and normal CRP possessed a lower level of radiographic damage, were less frequently seropositive for rheumatoid factor (RF) and anti-cyclic citrullinated peptide antibody (anti-CCP), and reported less usage of biologic disease-modifying antirheumatic drugs (bDMARDs) and glucocorticoids. Sex, age distribution, and methotrexate (MTX) administration were comparable between the two groups. As expected, subjective and composite remission rates defined by patient VAS ≤ 1 cm, HAQ-DI < 0.5, SDAI ≤ 3.3, and Boolean-based remission were greater in patients with RA with negative joint counts and normal CRP values than the remaining patients with RA (71.8% vs 29.2%, *p* < 0.0001; 78.5% vs 45.8%, *p* < 0.0001; 83.9% vs 16.8%, *p* < 0.0001; and 71.8% vs 16.1%, *p* < 0.0001; respectively).

### Comparison of the non-achievement rates of subjective clinical or functional remission among the different age groups between non-rheumatic patients and patients with RA in “objective clinical remission”

Rates of patient VAS > 1 cm and HAQ-DI ≥ 0.5 among the non-elderly, early elderly, and late-elderly patients without rheumatic diseases were compared with those of patients with RA in “objective clinical remission.” Notably, subjective non-achievement of remission criteria was almost exclusively observed in the late-elderly patients: 7.3% for patient VAS > 1 cm and 14.5% for HAQ-DI ≥ 0.5 (0.0–1.0% in non-elderly and early elderly patients, Table 2). In contrast to the above findings, > 20% and > 10% of patients with RA with negative joint counts and normal CRP values did not meet the patient VAS ≤ 1 cm and HAQ-DI < 0.5 criteria, respectively, even in patients aged < 75 years (early elderly or non-elderly). Furthermore, 34.0% and 35.8% of late-elderly patients with RA had VAS > 1 cm and HAQ-DI ≥ 0.5, respectively, despite “objective clinical remission.” Therefore, it was demonstrated that a considerable portion of RA patients had subjective/functional disturbance even in the absence of objective findings.

**Table 2:** Comparison of the non-achievement rates of subjective remission among different age groups of patients with and without rheumatic diseases.

Non-rheumatic patients (n = 301)	< 65 years (n = 97) (%)	65–74 years (n = 80) (%)	≥ 75 years (n = 124) (%)	*p* value
Patient VAS > 1 cm	1.0	0.0	7.3	0.0061
HAQ-DI ≥ 0.5	0.0	0.0	14.5	< 0.0001

RA patients in “objective clinical remission” (n = 149)	< 65 years (n = 48) (%)	65–74 years (n = 48) (%)	≥ 75 years (n = 53) (%)	*p* value
Patient VAS > 1 cm	22.9^a^	27.1^a^	34.0^a^	0.46
HAQ-DI ≥ 0.5	10.4^b^	16.7^c^	35.8^d^	0.0049

*p* values were calculated using the Fisher’s exact test for non-rheumatic patients and Pearson’s chi-square test for RA patients.

^a^*p* < 0.0001, ^b^*p* = 0.0034, ^c^*p* = 0.0003, ^d^*p* = 0.0023, via Fisher’s exact test as compared with non-rheumatic patients.

### Multivariate analysis of factors associated with the non-achievement of subjective remission within a combined population of non-rheumatic and patients with RA in the absence of objective findings

In order to evaluate whether age, sex, and the presence of RA is associated with the non-achievement of subjective clinical or functional remission, we combined the data from non-rheumatic patients and patients with RA with negative joint counts and normal CRP. Age showed marginal and significant impacts on non-achievement of VAS ≤ 1 cm (15.3% in late-elderly vs 9.2% in others; *p* = 0.051) and HAQ-DI < 0.5 (20.9% in late-elderly vs 4.8% in others; *p* < 0.0001), respectively. Sex was associated with HAQ-DI scores that were ≥ 0.5 (14.9% in female vs 5.1% in male patients; *p* = 0.0011), but not with patient VAS > 1 cm (13.5% vs 8.6%, respectively; *p* = 0.13). The presence of RA, even without objective findings, was significantly associated with patient VAS > 1 cm (28.2% in patients with RA without objective findings vs 3.3% in non-rheumatic patients; *p* < 0.0001) and HAQ-DI ≥ 0.5 (21.5% vs 6.0%, respectively; *p* < 0.0001). Because sex and RA are confounding (Table 1; Supplementary Table 1), a multivariate logistic analysis was subsequently performed to clarify the effects of the above parameters on the non-achievement of patient VAS ≤ 1 cm and HAQ-DI < 0.5 (Table 3). We confirmed that age and the presence of RA were significantly associated with the non-achievement of patient VAS ≤ 1 cm and HAQ-DI < 0.5, and that sex (female) was significantly associated with the non-achievement of HAQ-DI < 0.5.

**Table 3:** Multivariate logistic regression analysis determining risk factors of not achieving clinical or functional remission.

	Patient VAS > 1 cm	HAQ-DI ≥ 0.5
Age (year)	1.029 (95% CI 1.004–1.055)	1.095 (95% CI 1.058–1.134)
Sex (female)	1.119 (95% CI 0.559–2.237)	2.843 (95% CI 1.289–6.271)
RA	11.913 (95% CI 5.675–25.006)	4.912 (95% CI 2.499–9.655)

*95% CI* 95% confidence interval.

## Discussion

To the best of our knowledge, this is the first study that examined patient VAS values and HAQ-DI scores with respect to musculoskeletal symptoms in 301 patients without rheumatic disease to compare them with those from patients with RA lacking any objective findings such as tender/swollen joints or elevated serum CRP values. In this study, we demonstrated that advanced age clearly affected the achievement of RA remission criteria irrespective of the presence of RA. Therefore, current assessments of subjective disease activity and functional state by patient VAS and HAQ-DI may not be effective in late-elderly patients. Moreover, the presence of RA itself, without any objective disease activity, was associated with worsened patient VAS and HAQ-DI scores.

It should be noted that the age distribution for patients with RA with negative joint counts and normal CRP values and remaining patients with RA examined were similar (Table 2), while radiographic damage and seropositivity, both of which are poor prognostic factors of RA^12^, were significantly different between the two groups (Supplementary Table 1). Thus, the effect of advanced age on the achievement of RA remission criteria is largely subjective, as indicated previously^7^, and this fact is likely to be related to differences between patient VAS and physician (evaluator) VAS scores^4,13^. The effect of age on RA outcome has previously been identified in a cohort of patients with early RA, the ESPOIR cohort (the French multicenter prospective cohort of patients with early arthritis)^14^. Additionally, it is important to note the difficulty in meeting RA remission criteria for patients in a general population of elderly individuals without rheumatic diseases^8^, as well in patients with RA associated with comorbidities^15–17^. Therefore, the inclusion of PRO in RA disease activity measures and corresponding remission criteria can be reconsidered in the future, as its usefulness for evaluating advanced-aged patients is diminished relative to younger ones^18–21^.

The effect of sex on HAQ-DI but not on patient VAS in this study may be an issue requiring investigation in the future. In a separate analysis of non-rheumatic and patients with RA, patient VAS > 1 cm was observed in 3.7% of male and 3.0% of female non-rheumatic patients (*p* = 0.76) and in 25.6% of male and 29.1% of female patients with RA (*p* = 0.84), while HAQ-DI ≥ 0.5 was observed in 3.7% of male and 7.9% of female non-rheumatic patients (*p* = 0.15) and in 10.3% of male and 25.5% of female RA patients (*p* = 0.068).

In this study, patients without rheumatic diseases completed a single global assessment of musculoskeletal symptoms (patient VAS for non-rheumatic patients), while patients with RA completed a global assessment of disease activity (patient VAS for RA patients) and a global assessment of pain (pain VAS). As expected, the pain VAS and patient VAS were strongly correlated (r^2^ = 0.843, *p* < 0.0001) even among RA patients in objective clinical remission. Therefore, our results indicate that a considerable portion of people aged ≥ 75 years without any diagnosis of rheumatic diseases suffer from musculoskeletal pain.

The limitations of our study are that the suitability of patient VAS for patients without rheumatic diseases is unknown, and the lack of CRP and radiographic joint evaluation data of those patients.

In conclusion, we clarified the importance of advanced age, which was independent of the presence of RA even without any objective disease activity, on PRO such as patient VAS and HAQ-DI scores, which should be increasingly important as human global populations are rapidly ageing.

## Methods

### Patients

Patients without rheumatic disease who visited a general medical clinic (Aoki Clinic) between September 2017 and December 2018 were considered. The patients gave informed consent and received a systemic, 68-joint examination to confirm the absence of an active rheumatic disease such as RA. All patients (n = 302) visiting between this time period except one, who had active arthritis, were enrolled in the study.

As a disease control, patients with RA (n = 149) with neither tender nor swollen joints and with normal serum CRP values (≤ 0.3 mg/dl), characteristics of “objective clinical remission,” were selected from the data of 304 patients with RA obtained from the Toho University Ohashi Medical Center between May 2014 and March 2015^7^. All patients met the ACR 1987 revised criteria^22^ and/or the ACR/EULAR 2010 classification criteria^23^ for RA.

The study protocol was approved by the Ethics Committee of Toho University Ohashi Medical Center (project approval number: H17028). All methods were performed in accordance with the Declaration of Helsinki and other relevant guidelines and regulations.

### Clinical assessments

Patients without rheumatic diseases completed a global assessment (0–10 cm visual analog scale) of musculoskeletal symptoms (patient VAS for non-rheumatic patients) and an HAQ-DI functional assessment. We obtained the following medical information from the medical records of non-rheumatic patients: age, sex, and disease diagnosis. For RA patients, records of age, sex, disease duration from the onset of symptoms to study enrolment, radiographic stage, global assessment of disease activity as measured by a 10-cm patient VAS, HAQ-DI, physician-based assessments of the presence of tenderness and swelling in 68 and 66 joints, respectively, serum levels of CRP, rheumatoid factor (RF), anti-cyclic citrullinated peptide antibody (anti-CCP), and treatment regimens were obtained. Physician-based assessment of the presence of tenderness and swelling in 68 and 66 joints, respectively, was used to screen to exclude patients demonstrating active rheumatic diseases such as RA and osteoarthritis from the patient group that did not have rheumatic disease.

### Statistical analysis

Statistical analysis was performed using JMP Pro (version 14.2.0, SAS Institute Japan Ltd. Tokyo, Japan). Continuous variables were summarized using the median and interquartile range (IQR) and were analyzed using the Mann–Whitney U test or the Kruskal–Wallis test. Binominal data between two or three groups were evaluated using Pearson’s chi-square test, and Fisher’s exact test was used where appropriate. A multivariate logistic regression analysis was performed to examine the conditional effects of clinical parameters on the achievement of patient VAS ≤ 1 cm or HAQ-DI < 0.5. *p* values < 0.05 were considered statistically significant.

## Supplementary information

Supplementary Table 1.

## Data Availability

The datasets generated during the current study are available from the corresponding author on reasonable request.
