# Supplementary material for: Association of age with the non-achievement of clinical and functional remission in rheumatoid arthritis
Source: Sci Rep. 2020 Sep 17;10:15277. doi: 10.1038/s41598-020-72274-2 (PMC7498589; doi:10.1038/s41598-020-72274-2)
Supplement: Supplementary file 1 — Supplementary Table 1. [file 41598_2020_72274_MOESM1_ESM.docx]

**Association of age with the non-achievement of clinical and functional remission in rheumatoid arthritis**

Tadashi Aoki, Hideki Ito, Takehisa Ogura, Ayako Hirata, Yuji Nishiwaki and Hideto Kameda

**Supplementary Table 1. Demographic features of RA patients with negative joint counts and normal CRP values.**

| Variables | RA patients with negative joint counts and normal CRP  (n = 149) | Other RA patients (n = 155) | p-value |
| --- | --- | --- | --- |
| Sex (female); n (%) | 110 (73.8) | 126 (81.3) | 0.12^a^ |
| Age (years); median (IQR) | 72 (60–79) | 71 (57–78) | 0.77^b^ |
| < 65 years, n (%) | 48 (32.2） | 56 (36.1） |  |
| 65–74 years, n (%) | 48 (32.2） | 38 (24.5) | 0.33^a^ |
| ≥ 75 years, n (%) | 53 (35.6） | 61 (39.4) |  |
| Disease duration (years); median (IQR) | 5.5 (2.6–9.0) | 6.0 (2.6–13.0) | 0.25^b^ |
| Steinbrocker’s radiographic stage (I or II); n (%) | 109 (77.3) | 77 (52.0) | < 0.0001^a^ |
| RF positive; n (%) | 87 (60.4) | 113 (74.8) | 0.0081^a^ |
| Anti-CCP positive; n (%) | 89 (64.0) | 113 (77.9) | 0.0098^a^ |
| Current MTX; n (%) | 94 (63.1) | 99 (63.9) | 0.89^a^ |
| Current bDMARDs; n (%) | 31 (20.8) | 59 (38.1) | 0.0010^a^ |
| Current glucocorticoids; n (%) | 19 (12.8) | 34 (21.9) | 0.035^a^ |
| Patient VAS ≤ 1 cm; n (%) | 107 (71.8) | 45 (29.2) | <0.0001^a^ |
| HAQ-DI < 0.5; n (%) | 117 (78.5) | 71 (45.8) | <0.0001^a^ |
| SDAI ≤ 3.3; n (%) | 125 (83.9) | 26 (16.8) | <0.0001^a^ |
| Boolean remission; n (%) | 107 (71.8) | 25 (16.1) | <0.0001^a^ |

Values are reported as the median (interquartile range; IQR) or number (%).

^a^Pearson’s chi-square test

^b^Mann–Whitney U test
